# Poster Session I - A62 INTESTINAL ULTRASOUND MEASUREMENT OF THE SUBMUCOSA BOWEL LAYER IN PEDIATRIC INFLAMMATORY BOWEL DISEASE: THICKNESS CUT-OFFS FOR IDENTIFYING INFLAMED BOWEL

**DOI:** 10.1093/jcag/gwaf042.062

**Published:** 2026-02-13

**Authors:** M Hong, E Chamseddine, M W Carroll, D M Isaac, H Q Huynh, A S Hudson

**Affiliations:** University of Alberta, Edmonton, AB, Canada; University of Alberta, Edmonton, AB, Canada; University of Alberta, Edmonton, AB, Canada; University of Alberta, Edmonton, AB, Canada; University of Alberta, Edmonton, AB, Canada; University of Alberta, Edmonton, AB, Canada

## Abstract

**Background:**

Intestinal ultrasound (IUS) total bowel wall thickness (BWT) correlates well with severity of endoscopic inflammatory bowel disease (IBD) activity. The bowel’s sub-layers are also of interest as additional clues for inflammation. In adults, increased total BWT (>3 mm) and submucosal thickness (SMT) to BWT ratio (>0.50) has been reported in active ulcerative colitis (UC). In children, a thinner total BWT cut-off (>2 mm) has been found for active IBD, but the SMT has not yet been reported.

**Aims:**

To assess the IUS measured SM and SM/total BWT ratio in pediatric patients with newly diagnosed CD and UC, to help inform cut-offs that distinguish normal vs. inflamed intestine.

**Methods:**

Pediatric patients (0-18 years) with suspected IBD were prospectively enrolled (between 2019-2024) and underwent baseline IUS, clinical assessment, blood work, calprotectin, and endoscopy. IUS included two total BWT measurements for bowel segment. For this study, IUS images were retrospectively reviewed and the SMT was measured within 0.5 cm of the original total BWT measurements using DiCOM viewer. Statistical analysis included descriptive, Spearman rho correlations, Mann-Whitney U group comparisons, and ROC curve analyses.

**Results:**

Eight-six patients (n = 431 intestinal segments) were included (n = 55, 64% male), with a median age at diagnosis of 13 years (IQR 11-15, range 3-17), majority with CD (n = 49, 57%). There were significant moderate correlations between endoscopic disease severity and thickness of both the total BWT and SMT for CD (rho=0.58 BWT, rho=0.49 SMT) and ulcerative colitis (rho=0.57 BWT, rho=0.54 SMT) (p < 0.001). SM/BWT ratio weakly correlated with endoscopy in UC (rho=0.27, p < 0.001), and didn’t significantly correlate in CD (rho=0.12, p = 0.09). The median SM was significantly thicker in inflamed intestine (1.1 mm CD, 1.4 mm UC) compared to normal intestine (0.8 mm CD, 0.8 mm UC) (p < 0.001). The optimal cut-off for detecting endoscopic inflammation was a SM ≥ 1.3 mm (for CD) (AUC 0.67, 95^th^ CI 0.59-0.74, p < 0.001) and ≥ 1.1 mm (for UC) (AUC 0.79, 95^th^ CI 0.72-0.86, p < 0.001). The optimal SM/BWT ratio cut-off was ≥0.55 for UC (AUC 0.65, 95^th^ CI 0.56-0.74, p = 0.001).

**Conclusions:**

Thickening of the SM bowel wall layer was found to be a new helpful indicator of active pediatric IBD, correlating well to endoscopic disease activity. For children, a SM thicker than 1 mm detected active IBD. A SM/BWT ratio >0.55 detected UC inflammation, but was not significant for CD. Possible explanations could be a more uniform thickening of the bowel wall in active CD compared to UC, but future research is needed.

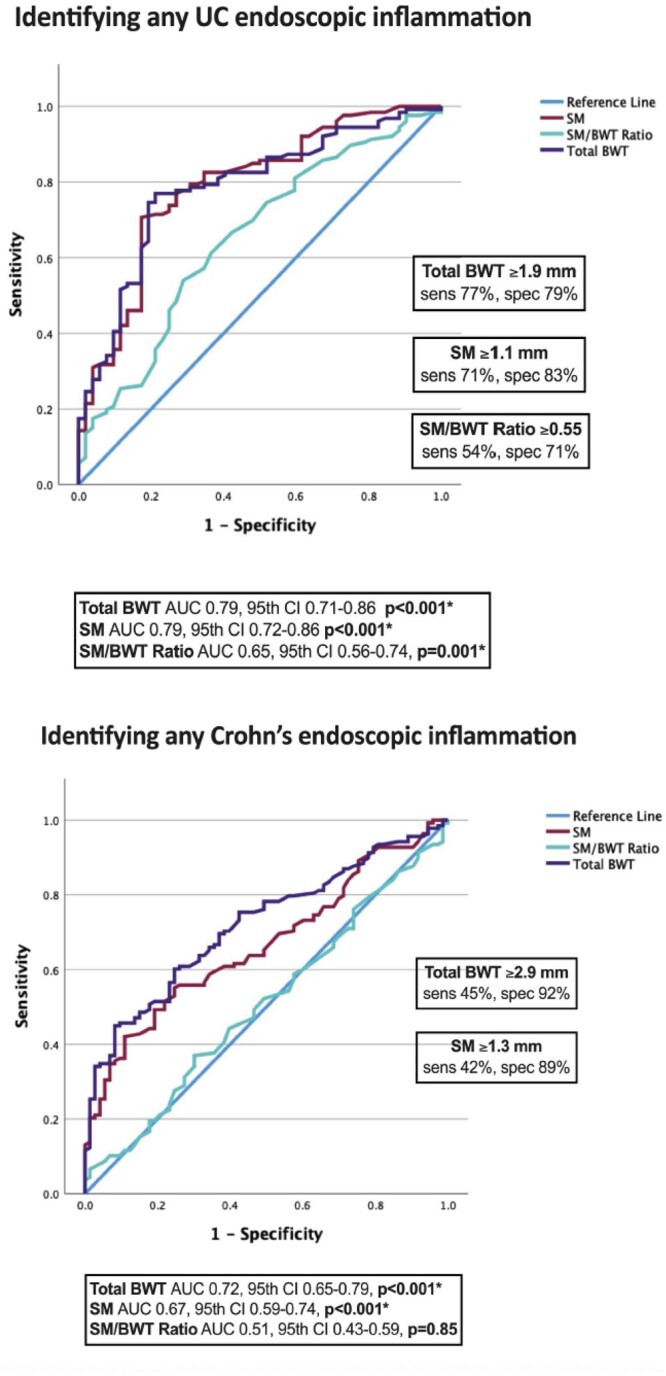

**Funding Agencies:**

North American Society For Pediatric Gastroenterology, Hepatology & Nutrition (NASPGHAN); Department of Pediatrics, University of Alberta, Canada

